# Motor Function of the Upper-Extremity after Transection of the Second Thoracic Nerve Root during Total En Bloc Spondylectomy

**DOI:** 10.1371/journal.pone.0109838

**Published:** 2014-10-15

**Authors:** Noriaki Yokogawa, Hideki Murakami, Satoru Demura, Satoshi Kato, Katsuhito Yoshioka, Hiroyuki Hayashi, Takayoshi Ishii, Moriyuki Fujii, Takashi Igarashi, Hiroyuki Tsuchiya

**Affiliations:** Department of Orthopaedic Surgery, Graduate School of Medical Sciences, Kanazawa University, Takara-machi, Kanazawa, Japan; Sainte-Anne Hospital Center, France

## Abstract

**Background:**

In total en bloc spondylectomy (TES) of upper thoracic spine including the second thoracic (T2) vertebra, T2 nerve roots are usually transected. In this study, we examined the association between transection of the T2 nerve roots and upper-extremity motor function in patients with upper thoracic TES.

**Methods:**

We assessed 16 patients who underwent upper thoracic TES with bilateral transection of the T2 nerve roots. Patients were divided into three groups: 3 patients without any processing of T1 and upper nerve roots (T2 group), 7 with extensive dissection of T1 nerve roots (T1–2 group), and 6 with extensive dissection of T1 and upper nerve roots (C–T2 group). Postoperative upper-extremity motor function was compared between the groups.

**Results:**

Postoperative deterioration of upper-extremity motor function was observed in 9 of the 16 patients (56.3%). Three of the 7 patients in the T1–2 group and all 6 patients in the C–T2 group showed deterioration of upper-extremity motor function, but there was no deterioration in the T2 group. In the T1–2 group, 3 patients showed mild deterioration that did not affect their activities of daily living and they achieved complete recovery at the latest follow-up examination. In contrast, severe dysfunction occurred frequently in the C–T2 group, without recovery at the latest follow-up.

**Conclusions:**

The transection of the T2 nerve roots alone did not result in upper-extremity motor dysfunction; rather, the dysfunction is caused by the extensive dissection of the T1 and upper nerve roots. Therefore, transection of the T2 nerve roots in upper thoracic TES seems to be an acceptable procedure with satisfactory outcomes.

## Introduction

**Figure 1:**
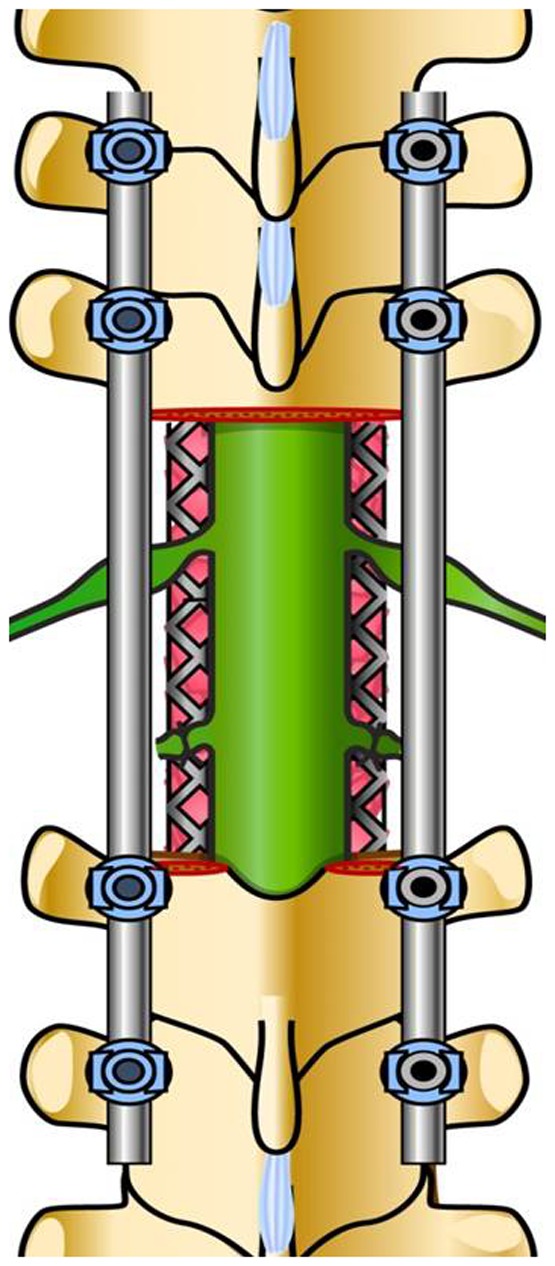
A schema of upper thoracic total en bloc spondylectomy; the T1 nerve roots are circumferentially dissected to the extraforamen and the T2 nerve roots are transected.

Total en bloc spondylectomy (TES) is a surgical procedure designed to achieve complete resection of spinal tumors, including primary malignant, aggressive benign, and metastatic tumors [1], [2]. Since 1989, we have performed more than 300 cases of TES at our institution, and our intervention with TES has decreased local tumor recurrence and improved patient prognoses [3], [4], [5]. In TES, en bloc resection of the posterior element (en bloc laminectomy) and en bloc resection of the anterior element (en bloc corpectomy) were performed to obtain an adequate tumor margin, with extensive dissection and/or transection of spinal nerve roots. In our surgical method for upper thoracic TES, in order to prevent upper-extremity motor dysfunction, the T1 and upper nerve roots are preserved by extensive circumferential dissection to the extraforamen, and the T2 and lower nerve roots are sacrificed by transection (Figure 1). Although the brachial plexus is mainly composed of the C5–T1 nerve roots, a contribution by the T2 nerve roots to the brachial plexus has been recently reported [6]; therefore, the postoperative deterioration of upper-extremity motor function due to transection of the T2 nerve root is a matter of concern. In this study, we examined the association between transection of the T2 nerve roots and upper-extremity motor function in patients with upper thoracic TES.

## Patients and Methods

### Ethics Statements

This study was approved by the ethics committee of Kanazawa University, and written informed consent for the surgery and entry into the research study was obtained from all patients.

### Patient Characteristics

We reviewed 170 patients who had undergone TES at our institution between January 2005 and January 2014 where data collection was possible, and identified 16 patients who had undergone upper thoracic TES with bilateral transection of the T2 nerve roots. We performed a retrospective review of the prospectively collected data for the patients. The study population included 7 men and 9 women, with a mean age of 52.8 years (range, 26–71 years) at the time of surgery. Of the 16 patients, 12 had metastatic tumors and 4 had primary tumors.

### Evaluation Item

Patients were divided into three groups: 3 patients without any processing of T1 and upper nerve roots (T2 group), 7 with extensive dissection of T1 nerve roots (T1–2 group), and 6 with extensive dissection of T1 and upper nerve roots (C–T2 group). Postoperative upper-extremity motor function was compared between the groups. Using the Japanese Orthopaedic Association (JOA) scoring system for the evaluation of cervical myelopathy (Table 1), scoring for upper-extremity motor function was performed 4 weeks after surgery and at the latest follow up; the mean follow-up period was 30.2 months (range, 1–67 months). The preoperative JOA score was normal in all patients.

**Table 1:** Japanese Orthopaedic Association (JOA) scoring system for the evaluation of cervical myelopathy.

Grade	Upper-extremity motor function
**0**	Unable to feed oneself
**1**	Unable to handle chopsticks, able to eat with spoon
**2**	Able to handle chopsticks with much difficulty
**3**	Able to handle chopsticks with slight difficulty
**4**	Normal

### Statistical Analysis

Statistical evaluation was performed using Scheffe's F-statistics for multiple comparisons in SPSS Statistical Software, version 19 (SPSS, Chicago, IL, USA). Statistical significance was set at a P-value less than 0.05.

## Results

**Figure 2:**
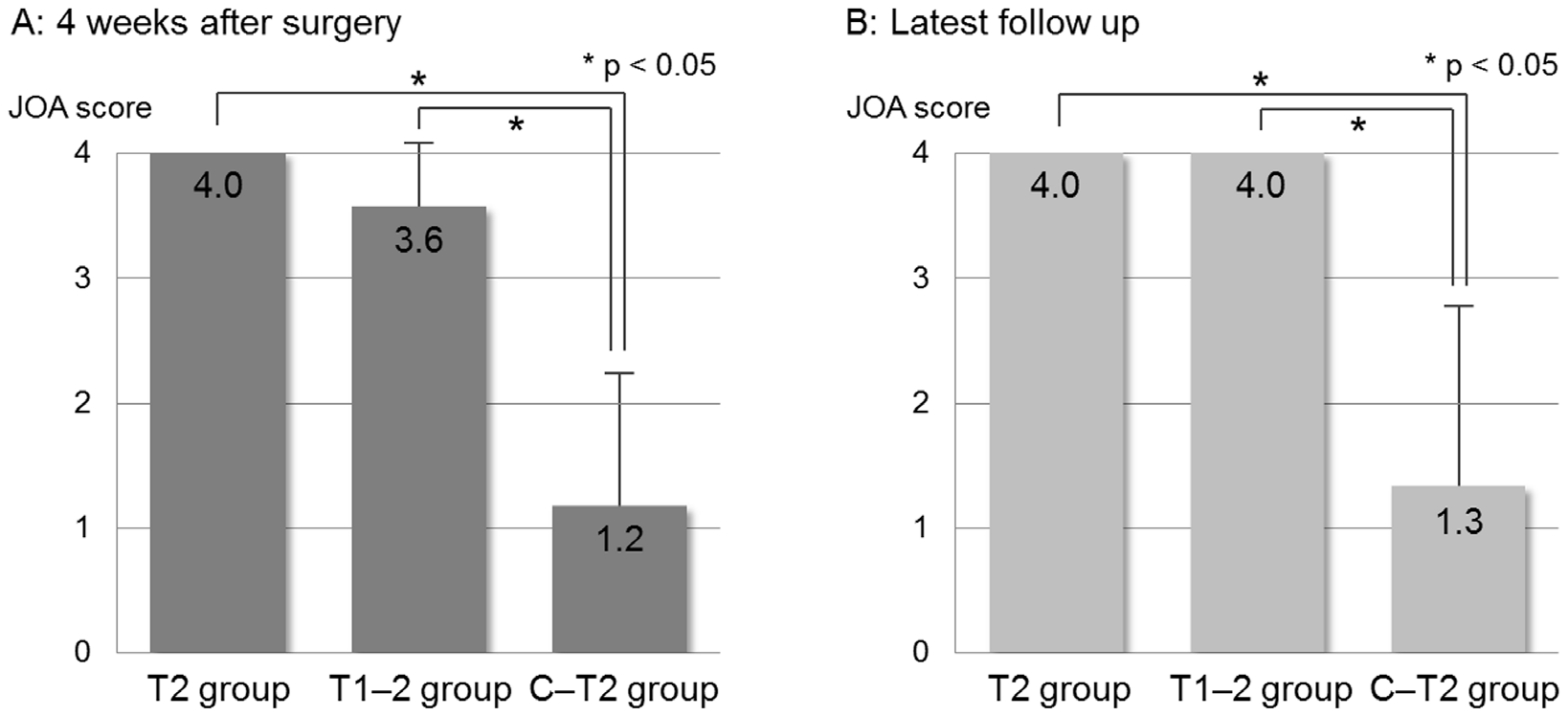
Postoperative mean Japanese Orthopaedic Association (JOA) scores (+ *SD*) 4 weeks after surgery (A) and at the latest follow up (B). Patients who underwent upper thoracic TES with bilateral transection of the T2 nerve roots were divided into three groups: no processing of T1 and upper nerve roots (T2 group, *n* = 3), extensive dissection of T1 nerve roots (T1–2 group, *n* = 7), and extensive dissection of T1 and upper nerve roots (C–T2 group, *n* = 6). The C–T2 group showed significantly more severe deterioration than the other two groups, both 4 weeks after surgery and at the latest follow up (P = 0.001).

Postoperative deterioration of upper-extremity motor function was observed in 9 of the 16 patients (56.3%). Three of 7 patients in the T1–2 group and all 6 patients in the C–T2 group showed deterioration of upper-extremity motor function, but there was no deterioration in the T2 group. Postoperative mean JOA scores 4 weeks after surgery and at the latest follow up were 4.0 and 4.0 in the T2 group, 3.6 and 4.0 in the T1–2 group, and 1.2 and 1.3 in the C–T2 group, respectively (Figure 2). The C–T2 group showed significantly more severe deterioration than the other two groups, both 4 weeks after surgery and at the latest follow up (P = 0.001). In the T1–2 group, all 3 patients showed mild deterioration that did not affect their activities of daily living, but they showed complete recovery at the latest follow-up examination. In contrast, severe dysfunction occurred frequently in the C–T2 group, with poor recovery at the latest follow-up. No patients showed postoperative neurological deterioration resulting from myelopathy.

## Illustrative Case Presentation

Written consent was obtained for the publication of these cases.

### Case 1 (T2 group)

**Figure 3:**
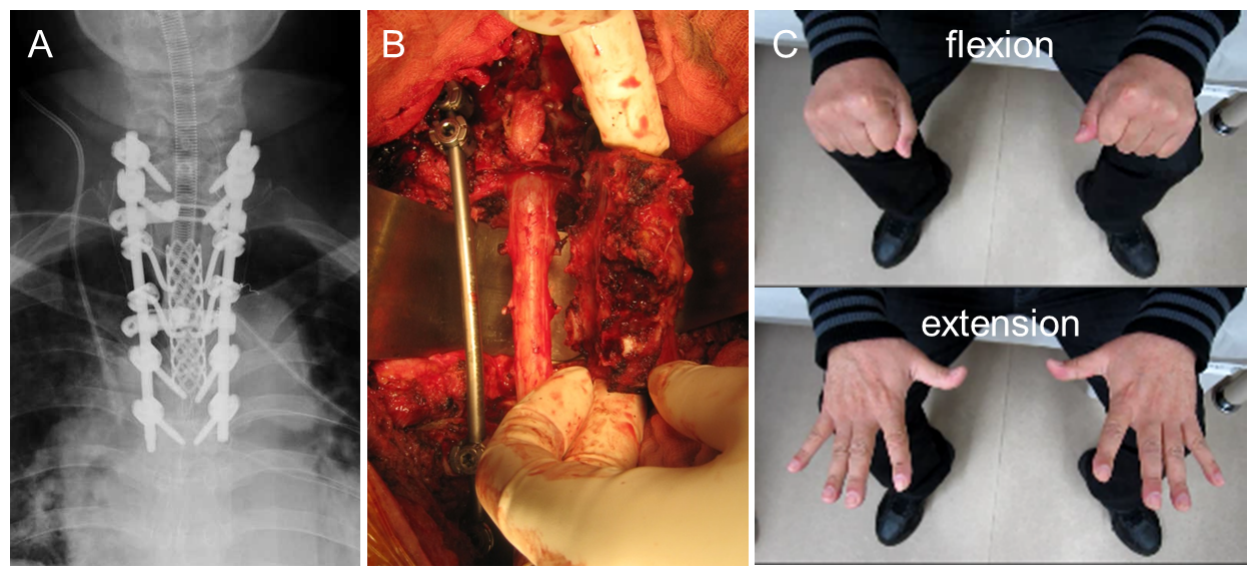
Case 1: A 49-year-old man with primary spinal tumor (angiosarcoma) at T2–4. A: Total en bloc spondylectomy (TES) was performed using a posterior-only approach. B: Bilateral T2–4 nerve roots were transected. C: No deterioration of upper-extremity motor function occurred after surgery.

A 49-year-old man with a primary spinal tumor (angiosarcoma) at T2–4 underwent TES via a posterior-only approach (Figure 3A). During the operation, bilateral T2–4 nerve roots were transected (Figure 3B). There was no deterioration of upper-extremity motor function after surgery (Figure 3C).

### Case 2 (C–T2 group)

**Figure 4:**
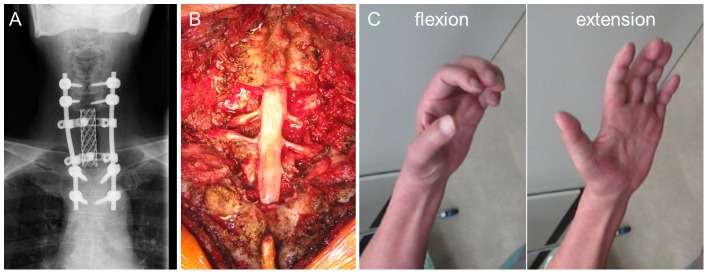
Case 2: A 71-year-old man with metastatic renal cell carcinoma at C7–T2. A: Total en bloc spondylectomy (TES) was performed using a combined anterior and posterior approach. B: Bilateral T2 nerve roots ware transected and bilateral C8–T1 nerve roots ware circumferentially dissected to the extraforamen. C: Severe upper-extremity motor dysfunction persisted even 3 years after surgery.

A 71-year-old man with metastatic renal cell carcinoma at C7–T2 underwent TES via a combined anterior and posterior approach (Figure 4A). During the operation, the bilateral T2 nerve roots ware transected and the bilateral C8–T1 nerve roots were circumferentially dissected to the extraforamen (Figure 4B). Severe upper-extremity motor dysfunction occurred, and he still could not feed himself due to severe paralysis 3 years after surgery (Figure 4C).

## Discussion

The structure of the brachial plexus has not been completely explained yet, even though it was originally described long ago, and recent studies still aim to elucidate the variation that has been observed in the contributions of nerve roots to the brachial plexus. Several studies have shown a contribution of the T2 nerve root to the brachial plexus [7], [8], and Loukas et al. have reported that 100% of the specimens they examined contained communicating branches between the T2 nerve root and the brachial plexus [9]. Thus, it is evident that the T2 nerve root has some relevance to the brachial plexus. However, it is not yet clear how the T2 nerve root makes this contribution, especially in association with upper-extremity motor function. To our knowledge, motor function of the T2 nerve root has not been previously reported clinically, because spinal nerve roots are usually preserved in spinal surgery to prevent the neurological deficits that result from transection. To resect a spinal tumor en bloc in TES, spinal nerve roots are necessarily sacrificed, and it is consequently possible to assess the function of these spinal nerve roots. However, patients where TES is indicated are limited and, in particular, TES of the upper thoracic level is very rare. Moreover, TES is performed at only a few institutions. Therefore, clinical data with a case series of T2 nerve root transections would be difficult to obtain at other institutions, and we believe that the current study, despite being retrospective and of a small sample size, contains highly valuable information.

The results of the current study show that transection of the T2 nerve root alone did not affect upper-extremity motor function. Although this suggests that the T2 nerve roots contribute little to upper-extremity motor function, we cannot draw that conclusion because there are individual variations in the communicating branch from the T2 nerve root to the brachial plexus [9], as well as the structure of the brachial plexus [10], [11]. Hence, further study must be conducted to elucidate the associations between T2 nerve roots and upper-extremity motor function. A thorough understanding can only be achieved if we increase the sample size significantly and acquire more accurate objective evidence using techniques such as electrophysiological testing.

Upper-extremity motor dysfunction occurred frequently in the patients who underwent extensive dissection of the T1 and upper nerve roots. This is probably due largely to the obstruction of blood flow. Spinal nerve roots are mainly supplied by the spinal branches, which are derived from the segmental arteries and flow into each nerve sheath at the exit of the foramen [12]. Hence, severe obstruction of blood flow may occur following extensive circumferential spinal nerve root dissection to the extraforamen, leading to deterioration of upper-extremity motor function. Furthermore, in a report on lumbar TES, postoperative muscle weakness of the lower-extremity was noted in patients after dissection of more than two levels of nerve roots [13]. Similarly, in the current study, severe upper-extremity motor dysfunction occurred in the patients who underwent dissection of more than two levels of nerve roots, including the T1 and upper nerve roots. Thus, it could be presumed that multiple spinal nerve roots dissection to the extraforamen is the risk factor for postoperative neurological deterioration, although there is insufficient evidence to prove this.

In conclusion, the transection of the T2 nerve roots alone did not result in upper-extremity motor dysfunction; rather the extensive dissection of the T1 and upper nerve roots was the cause of upper-extremity motor dysfunction. Therefore, transection of the T2 nerve roots in upper thoracic TES seems to be an acceptable procedure with satisfactory outcomes. Minimizing the dissection of the T1 and upper nerve roots can prevent postoperative upper-extremity motor dysfunction in upper thoracic TES. Additionally, in cases where the extensive dissection of nerve roots cannot be avoided, adequate informed consent is very important. The information from this study will be useful for deciding on an appropriate strategy for any surgery that involves processing of the brachial plexus, such as TES.
